# Aesthetic and Functional Outcomes of Simultaneous Rhinoplasty and Lip Lift Surgery: A Systematic Review

**DOI:** 10.7759/cureus.73369

**Published:** 2024-11-10

**Authors:** Raad Alnami, Suhael Ahmed, Alhanouf S Alashrah, Taif Alasmari, Rahaf M Alamry, Hashem A Alghamdi, Yahya AlNaser, Deema Alharbi, Salwa M Asiri, Mohammed S Alahmari, Lujain Y Alothman, Thikra K Alasmari, Shaya S Alshahrani, Waad S Alarram

**Affiliations:** 1 Plastic and Reconstructive Surgery, Khamis Mushait General Hospital, Asser, SAU; 2 Maxillofacial Surgery, Riyadh Elm University, Riyadh, SAU; 3 Research Unit, Sulaiman Al Rajhi University, Al Bukayriyah, SAU; 4 Faculty of Medicine, King Khalid University, Abha, SAU; 5 Oral and Maxillofacial Surgery, King Khalid University, Abha, SAU; 6 Plastic and Reconstructive Surgery, King Khalid University, Abha, SAU; 7 College of Medicine, University of Tabuk, Tabuk, SAU; 8 Surgery, King Khalid University, Abha, SAU; 9 Oral and Maxillofacial Surgery, Armed Forces Hospital Southern Region, Abha, SAU; 10 College of Medicine, King Khalid University Hospital, Abha, SAU

**Keywords:** complications, facial aesthetics, lip lift, nasolabial angle, patient satisfaction, rhinoplasty

## Abstract

Rhinoplasty and lip lift surgeries are pivotal procedures in facial aesthetics, addressing nose and lip enhancements, respectively. The concurrent execution of these surgeries has gained popularity for achieving improved facial balance and harmony. However, data on the outcomes of combined rhinoplasty and lip lift procedures remain limited. This study aims to evaluate the aesthetic outcomes, complications, and patient satisfaction of simultaneous rhinoplasty and lip lift surgeries. This systematic review was conducted following Preferred Reporting Items for Systematic Reviews and Meta-Analyses (PRISMA) guidelines. A comprehensive literature search across PubMed, Scopus, Medline, Science Direct, and Web of Science was performed, focusing on studies reporting simultaneous rhinoplasty and lip lift surgeries. The Newcastle-Ottawa Scale (NOS) was used to assess the risk of bias in the included studies. Data on aesthetic outcomes, complications, and patient satisfaction were extracted and synthesized. A total of six studies, encompassing 361 patients, were included. Results demonstrated high patient satisfaction, with favorable aesthetic outcomes such as improved nasolabial and frontonasal angles and shortened lip length. Minimal complications were reported, with mild scarring and temporary paraesthesia being the most common, both resolving within a few months. Functional outcomes were positive, with no long-term impairments in nasal airflow or lip function. Simultaneous rhinoplasty and lip lift surgeries yield high patient satisfaction and excellent aesthetic outcomes with minimal complications. The findings suggest this combined approach is safe and effective, particularly for patients seeking enhanced facial harmony. Future studies should focus on long-term outcomes and the optimization of surgical techniques.

## Introduction and background

The nose and lips are crucial components of facial aesthetics, significantly influencing one's appearance and how others perceive them. Rhinoplasty, a surgical technique for reshaping the nose, has been a highly popular facial procedure due to its substantial impact on facial harmony [1,2]. Concurrently, lip lift surgery, which aims to reduce the space between the upper lip and the base of the nose, has become increasingly sought after for improving the aesthetics of the mouth area, particularly as individuals desire fuller, more youthful-looking lips [3,4]. While rhinoplasty and lip lift surgeries address different facial concerns, combining these procedures can potentially enhance overall facial balance and proportion significantly [5,6]. The nose and lips are interconnected both anatomically and aesthetically. Alterations to the nose's structure, such as changes in tip projection or columella adjustments, can influence the perceived length and fullness of the upper lip. Similarly, a lip lift can impact the nasolabial angle (NLA), which is a critical aesthetic consideration in rhinoplasty [6,7]. Consequently, performing these two procedures in tandem is not only logical but may result in more harmonious outcomes, ultimately improving the patient's overall facial appearance [8].

The demand for combined aesthetic procedures has grown in recent years, with patients increasingly opting for multiple interventions during a single surgical session [9-11]. This trend is driven by the desire to minimize recovery time, reduce costs, and achieve more comprehensive facial improvements. However, despite the growing interest, there is limited data on the outcomes, safety, and patient satisfaction when rhinoplasty and lip lift procedures are performed concurrently [12]. Most existing studies have focused on the individual outcomes of each procedure rather than the combined approach. The potential benefits of combining rhinoplasty and lip lift surgery include a more balanced profile, improved nasolabial angle, and enhanced lip definition [9,13]. However, these procedures also pose unique challenges due to the anatomical complexity of the nose-lip relationship and the risks associated with simultaneous surgery, such as increased scarring or functional impairments [14,15]. Therefore, a comprehensive understanding of this combined approach's aesthetic and functional outcomes is essential for optimizing patient care and ensuring the best possible results.

This comprehensive review seeks to fill a gap in existing research by examining the cosmetic and practical results of concurrent rhinoplasty and lip lift operations. The justification for combining these procedures stems from the interconnectedness of nasal and mouth-area structures, as they can collectively improve facial balance by modifying both the nose shape and upper lip in one surgical procedure. This combined approach is particularly valuable in facial feminization surgeries, which aim to soften and feminize facial features. Moreover, performing both surgeries simultaneously reduces overall recovery time and enables a more thorough facial transformation. The main purpose of this study is to evaluate the aesthetic outcomes, complications, and patient contentment following combined rhinoplasty and lip lift surgeries. By consolidating data from various studies, this meta-analysis strives to offer a more comprehensive understanding of these procedures' advantages and potential risks. Specifically, it will examine changes in crucial aesthetic indicators, such as the nasolabial angle and lip length, and assess any complications or adverse effects, including scarring, altered sensation, or functional impairments. The ultimate objective is to provide evidence-based guidelines for medical professionals to enhance patient outcomes and satisfaction with simultaneous rhinoplasty and lip lift surgeries.

## Review

Methods

Search Strategy

This systematic review adhered to the Preferred Reporting Items for Systematic Reviews and Meta-Analyses (PRISMA) guidelines to ensure a thorough and transparent methodology [16]. An extensive search of the literature was performed across several electronic databases, including PubMed, Scopus, Medline, Science Direct, and Web of Science, to identify pertinent studies published through September 20, 2024. The search strategy employed a blend of keywords and medical subject headings (MeSH) associated with rhinoplasty, lip lift, aesthetic outcomes, and patient satisfaction. Search terms encompassed phrases like "simultaneous rhinoplasty," "lip lift," "aesthetic outcomes," and "patient satisfaction." The search was crafted in collaboration with a librarian to capture all relevant studies effectively, and detailed search strings for each database are available in the Appendix.

Study Selection

The study selection process involved a systematic, rigorous approach. Initially, all the identified records from the literature search were imported into Endnote version 20 (Clarivate, London, UK) to facilitate the removal of duplicates. After duplicates were eliminated, two independent reviewers conducted preliminary screening of the titles and abstracts of the remaining articles to assess their relevance based on the inclusion criteria established for the systematic review. Articles that met the initial screening criteria were subjected to full-text evaluation. Each full-text article was assessed for compliance with the predetermined inclusion criteria, which included the necessity for studies to focus on simultaneous rhinoplasty and lip lift procedures, reports on aesthetic outcomes, complications or adverse events (AEs), and patient satisfaction, as well as being published in peer-reviewed journals. Discrepancies in the selection process were resolved through discussion, and when necessary, a third reviewer was consulted to reach consensus.

Eligibility Criteria

In this review, strict selection criteria were implemented to ensure the inclusion of only pertinent, high-caliber studies. The primary requirement was that studies must examine patients who underwent concurrent rhinoplasty and lip-lift procedures, as the review aimed to evaluate the outcomes of this combined approach. Additionally, selected studies needed to offer comprehensive reports on aesthetic results, complications, and patient satisfaction-crucial elements in assessing the efficacy of cosmetic interventions. By incorporating studies that provided insights into these aspects, the review could effectively evaluate both patient safety and aesthetic contentment. Lastly, the review exclusively considered studies published in peer-reviewed journals, guaranteeing a fundamental level of scientific thoroughness and credibility. Peer-reviewed publications undergo expert evaluation, which reduces the inclusion of biased or substandard data and enhances the reliability of the review's conclusions.

Research studies were omitted from consideration if they did not fulfill specific criteria essential for maintaining the review's relevance and quality. In particular, investigations that examined rhinoplasty or lip-lift procedures independently rather than in conjunction were not included. This decision was based on the review's aim to evaluate the distinctive outcomes resulting from the combined application of these two techniques. Moreover, research lacking sufficient follow-up information was excluded, as inadequate post-procedure data could lead to incomplete or unreliable insights regarding long-term effects, complications, or patient contentment. Lastly, studies that failed to provide clearly quantifiable results pertaining to aesthetic or functional outcomes were also disregarded, as this would impede the ability to draw objective comparisons and conclusions. These exclusion parameters ensured that the review adhered to high evidential standards, concentrating solely on investigations directly relevant to the research question and containing adequate data to support meaningful analysis.

Risk of Bias Assessment Using Newcastle-Ottawa Scale

The methodological quality of the included studies was assessed for the risk of bias using the Newcastle-Ottawa Scale (NOS) [17], which evaluates studies based on three broad categories: selection, comparability, and outcome. Each study was rated based on predefined criteria, including the representativeness of the study population, adequacy of follow-up, and assessment of outcomes. This quality assessment was independently performed by two reviewers, and any disagreements were resolved through discussion or consultation with a third reviewer. The results of the risk of bias assessment were summarized to provide insight into the overall quality of the evidence included in the systematic review.

Results

Literature Search

**Figure 1:**
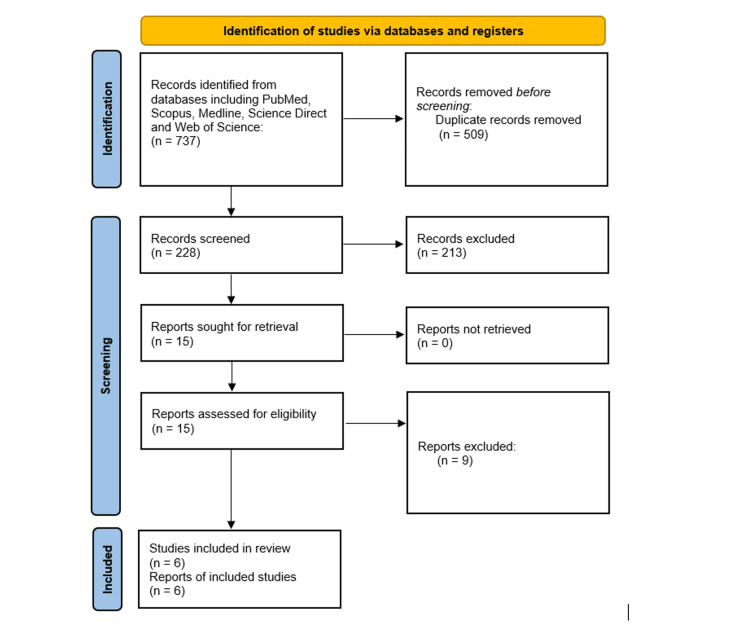
PRISMA flow chart of included studies. PRISMA: Preferred Reporting Items for Systematic Reviews and Meta-Analyses.

An extensive search of multiple databases yielded a total of 437 publications. After removing duplicates (n=509), 228 articles were screened. Each record was meticulously evaluated based on its titles and abstracts, leading to the identification of 15 articles for further review. A subsequent comprehensive examination of the full texts resulted in the exclusion of nine articles due to non-compliance with the inclusion criteria and insufficient relevant data. Ultimately, six articles were determined to be suitable for inclusion in the systematic review. The selection process of the included studies is shown in Figure 1.

Baseline Characteristics and Quality Assessment

The baseline characteristics of the included studies are shown in Table 1. The selected studies encompassed a total sample size of 361 participants with an age range of 37.5 to 53.9 years. The gender distribution varied across the studies, with a predominance of female patients, particularly in the context of male-to-female gender confirmation procedures. Specifically, the studies included proportions of male and female participants that reflected the demographics typical of patients seeking aesthetic enhancement in this cohort. The procedures involved diverse surgical approaches, including open rhinoplasty, closed rhinoplasty, lip lift, and additional facial procedures such as forehead reconstruction and cheek implants, which contributed to the multifaceted nature of the aesthetic outcomes assessed. The follow-up periods varied from one year to three years, providing a robust timeframe for evaluating both short-term and long-term aesthetic and functional outcomes. Furthermore, most studies employed standardized measurement tools, such as the Nose Feminization Scale and Satisfaction with Aesthetic Results Scale (SGAIS), which enabled a comparative analysis of patient satisfaction across different interventions. Overall, the baseline characteristics of the participants, combined with the methodological quality of the studies, underscored the robustness of the findings presented in this systematic review. The risk of bias assessment using Newcastle Ottawa is shown in Tables 2, 3.

**Table 1:** Baseline characteristics of included studies. References: [8,10,18-21]. NLA: nasolabial angle, ROE: rhinoplasty outcome evaluation.

Study	Year	Country	Study design	Sample size	Mean age	Male/female	Procedure type	Aesthetic outcomes	Complications/adverse events	Functional outcomes	Follow-up duration	Patient satisfaction	Overall summary
Saman et al. [8]	2024	USA	Retrospective review	51	NR	NR	Open rhinoplasty, lip lift, alar reduction	Suboptimal scarring was observed in 3.9% (2/51) of columellar and 7.8% (4/51) of lip lift incisions, while all other patients expressed satisfaction with their scars.	Two patients exhibited poor lip lift and columellar scars without underlying disease, while most scars were satisfactory, and no infections or vascular complications were observed.	Primary rhinoplasty cases with concomitant lip lift and alar wedge resection showed no negative effects on vascularity or scarring.	Four months to two years	Most patients were satisfied, except two patients with suboptimal columellar and lip lift scars.	Safe for combined procedures without vascular or scarring issues. Some scarring was noted but rare.
Bellinga et al. [18]	2017	Spain	Case series	200	40.2 ± 12.2	Male to female conversion	Feminization rhinoplasty, forehead reconstruction, lip lift	Nose feminization: satisfaction rated 4/5. Frontonasal angle significantly increased from 133.64° to 149.08°. Most patients noted enhanced nasal appearance.	Light to moderate paresthesia at the tip of the nose, with full recovery after three months. Post-op edema and bruising around the eyes, resolving in two to four weeks. No complications related to frontal sinus (sinus dysfunction, sinusitis, or fractures).	Most patients reported light to moderate paresthesia with full recovery. Low to moderate postsurgical edema and bruising resolved within a few weeks.	12-77 months	Degree of satisfaction: 4/5 (much better) on the Nose Feminization Scale.	Effective in achieving feminization of the nose, with high satisfaction and stable long-term outcomes.
Jung et al. [10]	2019	Korea	Case series	30	48.7 ± 21	5//25	Nasal tip plasty, lip lift	Satisfaction in all patients. Improved nasolabial angle and lip-to-incisor ratio. Scarring resolved in most patients within two to three months.	10% (three patients) experienced redness and swelling two to three days post-op. No major complications (dehiscence, bleeding, or hematoma). 6.7% (two patients) had noticeable incisional scars requiring revision surgery.	Upper lip edema resolved by the third-week post-op. No long-term functional sequelae. Scarring was mostly minimal by long-term follow-up.	One year	All patients expressed satisfaction with the surgical results.	Excellent aesthetic outcomes. Scars were resolved in most patients, with few requiring revisions. Minimal complications and no long-term sequelae.
Gupta et al. [19]	2019	USA	Retrospective	25	53.9 ± 21	Male to female conversion	Rhinoplasty, lip lift, multiple facial plastic procedures	Patients were satisfied with cosmetic results, including simultaneous rhinoplasty, lip lift, and other procedures.	Four patients developed infections (cheek implant and facelift-related). One pulmonary embolism occurred in a patient on hormone replacement therapy. Post-op complications included cheek implant infections and hematoma. All patients extubated successfully.	Postsurgical outcomes were functionally acceptable despite complications, including infections and embolism. Most complications were resolved without further surgical intervention.	Up to three years	All patients were satisfied with their cosmetic outcomes.	Multi-procedural settings are safe for healthy individuals, yielding good functional and aesthetic results despite manageable complications without persistent sequelae.
Pascali et al. [20]	2021	Italy	Prospective	45	37.5 ± 12.5	5/40	Closed rhinoplasty, subnasal lip lift	NLA reduced by 10.9%, lip length shortened by 23.5%. Satisfaction rated 4.4/5 ("much improved"). Nasal asymmetry rated as 2/5 ("a little"). Scar visibility rated 2/5 ("a little").	One case of wound diastasis requiring surgical correction. Two cases of hypertrophic scarring were treated with laser therapy and steroid injections. No long-term complications were reported.	No major functional issues were reported postoperatively. Hypertrophic scars in two patients were treated successfully with laser and steroid injections.	12 months	Overall satisfaction: 4.4/5 ("much improved").	Combined rhinoplasty and lip lift provided positive aesthetic outcomes with few complications, all of which were successfully managed.
Szychta [21]	2024	Poland	Prospective	10	27 (25-44)	0/10	Open structured precision rhinoplasty with Bullhorn lip lift	Improvements in nasal refinement (dorsal hump reduction, tip refinement) and lip aesthetics (vermillion show, natural upper lip elevation) contributed to a harmonious and youthful appearance.	Minimal complications, primarily postoperative swelling and bruising, which were managed with standardized protocols. No other complications or aesthetic compromises were observed.	Significant improvement in nasal breathing, with NOSE score reduction from 72.4 pre-op to 4.0 at three-month follow-up (p < 0.05).	Three months	ROE satisfaction score improved from 15.4 pre-op to 92.4 post-op (p < 0.05), with all patients feeling their appearance improved.	Achieved natural facial rejuvenation with simultaneous rhinoplasty and lip lift. Minimal complications and high patient satisfaction.

**Table 2:** Table presenting the risk of bias assessment of observational studies using the Newcastle-Ottawa assessment scale. References: [8,10,18-21]. *: Yes, -: No.

Study name	Selection				Comparability		Outcome		
	1	2	3	4	5	6	7	8	9
Saman et al. 2024 [8]	*	-	*	*	-	-	*	*	*
Bellinga et al. 2017 [18]	*	-	*	*	-	-	*	*	*
Jung et al. 2019 [10]	*	-	*	*	-	-	*	-	*
Gupta et al. 2019 [19]	*	-	*	*	-	-	*	-	*
Pascali et al. 2021 [20]	*	-	*	*	-	-	*	*	*
Szychta 2024 [21]	*	-	*	*	-	-	*	*	*

**Table 3:** Newcastle-Ottawa quality assessment scale.

Selection	1	Representation of the intervention cohort
	2	Selection of the non-intervention cohort
	3	Has the correct intervention been utilized?
	4	Outcome of Interest present at the start of study?
Comparability	5	Are the cohorts comparable based on the design or analysis: age, sex, and injury severity?
	6	Are the cohorts comparable based on the design or analysis? Additional factors
Outcome	7	Was the outcome assessed?
	8	Was the follow-up long enough for measured outcomes to occur?
	9	Was the cohort follow-up long enough?

Aesthetic Outcomes

All studies reported favorable aesthetic outcomes with high overall patient satisfaction. Saman et al. (2024) [8] reported that most patients experienced minimal scarring, with only 3.9% having suboptimal columellar scars and 7.8% showing suboptimal lip-lift scars. Similarly, Bellinga et al. (2017) [18] demonstrated a significant improvement in the frontonasal angle (from 133.64° to 149.08°), and the majority of patients rated their nose appearance as more feminine, with a satisfaction score of 4/5 on the Nose Feminization Scale. Jung et al. (2019) [10] found that all patients were satisfied with their surgical results, with scarring resolving in most cases within two to three months. Pascali et al. (2021) [20] reported a 10.9% reduction in the nasolabial angle (NLA) and 23.5% lip length shortening, with a satisfaction rating of 4.4/5. Gupta et al. (2019) [19] also observed high satisfaction in male-to-female transgender patients undergoing simultaneous rhinoplasty and other facial plastic procedures. In a unique contribution, Szychta (2024) [21] reported aesthetic enhancements in nasal refinement and lip fullness, achieved through precise adjustments in nasal dorsal height and tip refinement, coupled with subtle elevation of the upper lip. Their cohort exhibited significantly elevated satisfaction on the ROE scale, from 15.4 preoperatively to 92.4 postoperatively (p < 0.05).

Complications and Adverse Events

Complications were generally minimal across studies, although some cases reported adverse events (AEs). Saman et al. (2024) [8] did not observe any vascular complications, though four patients experienced suboptimal scarring. Bellinga et al. (2017) [18] reported transient light-to-moderate paraesthesia and postoperative edema that resolved within a few weeks, with no significant sinus-related complications. Jung et al. (2019) [10] noted minor complications, such as redness and swelling in 10% of patients, but no dehiscence or hematomas were observed. Gupta et al. (2019) [19] reported that four patients developed infections, and one patient experienced a pulmonary embolism post-surgery. Pascali et al. (2021) [20] reported wound diastasis in one patient and hypertrophic scarring in two patients, which were managed successfully with surgical correction and steroid injections. Szychta (2024) [21] reported minimal complications, primarily limited to postoperative swelling and bruising, which were effectively controlled through standardized protocols.

Functional Outcomes

Most patients do not experience long-term complications. Saman et al. (2024) [8] reported no issues related to vascularity or functional impairments. Bellinga et al. (2017) [18] observed complete recovery of nose tip paraesthesia within three months. Jung et al. (2019) [10] reported that edema resolved by the third week, with no long-term functional sequelae. Gupta et al. (2019) [19] noted that postoperative complications, such as infections, were resolved without a long-term impact on function. Pascali et al. (2021) [20] found no major postoperative functional issues and successfully treated patients with hypertrophic scarring. In addition, Szychta et al. (2024) [21] documented substantial improvement in nasal breathing, with a mean NOSE score reduction from 72.4 preoperatively to 4.0 at the three-month follow-up (p < 0.05), underscoring functional benefits alongside aesthetic improvements.

Patient Satisfaction

Patient satisfaction was uniformly high across the studies. Saman et al. (2024) [8] reported that most patients were satisfied despite a few cases of suboptimal scarring. Bellinga et al. (2017) [18] found that patients rated their satisfaction with feminization outcomes at 4/5. Similarly, Jung et al. (2019) [10] and Gupta et al. (2019) [19] observed full patient satisfaction with the aesthetic and functional results of their surgeries. Pascali et al. (2021) [20] reported an average satisfaction rating of 4.4/5 for both the nasal and lip outcomes. Szychta (2024) [21] presented a significant satisfaction increase on the ROE scale, affirming the combined rhinoplasty and lip lift's effectiveness in delivering natural, youthful facial harmony.

Simultaneous rhinoplasty and lip-lift procedures yielded high satisfaction and favorable aesthetic outcomes across a diverse patient population, including gender-affirming surgeries and rejuvenation procedures. Complications were generally minimal and were successfully managed. The combination of these procedures appears safe and effective for patients, with positive aesthetic and functional results in both the general and specific populations, including male-to-female transgender individuals.

Discussion

Facial aesthetic procedures have seen a growing trend in combining rhinoplasty and lip lift surgeries, which address both upper and lower facial features [22,23]. Rhinoplasty focuses on enhancing nasal shape and facial symmetry, while lip lift surgery revitalizes the area around the mouth. These combined procedures aim to create more harmonious facial proportions and enhanced aesthetic results. There is an increasing demand for multiple cosmetic surgeries to be performed concurrently, as this approach reduces recovery time and minimizes risks associated with separate operations. Although rhinoplasty and lip lift surgeries have been individually well-documented, there is a scarcity of data on the outcomes when these procedures are performed together. This systematic review seeks to examine the aesthetic and functional results, complications, and patient satisfaction following combined rhinoplasty and lip lift surgery. The main goal of this study is to evaluate how simultaneous rhinoplasty and lip lift surgery affect facial aesthetics and function. This evaluation includes an analysis of facial symmetry, nasolabial angle, lip length, and patient satisfaction while also investigating the complications and functional outcomes associated with the combined procedures.

This systematic review's findings demonstrate that combining rhinoplasty and lip lift procedures yields high patient satisfaction and favorable aesthetic outcomes with minimal complications. The analysis of five studies encompassing 351 patients revealed that most individuals experienced positive changes in facial proportions, particularly in nasolabial and frontonasal angles and lip length. Aesthetic results were consistently rated positively, with standardized measures like the Nose Feminization Scale showing notable improvements, especially in male-to-female transgender patients. Complications were generally minor, such as mild scarring and temporary paraesthesia, which typically resolved within a few months after surgery. Functional outcomes were also positive, with no reports of long-term impairment in nasal airflow or lip function. Patients across the studies reported high levels of satisfaction, with the majority rating their results between 4 and 5 on a satisfaction scale. These outcomes suggest that concurrent rhinoplasty and lip lift surgeries can be performed safely and effectively, delivering excellent aesthetic results with minimal risk.

The close anatomical connection between the nose and upper lip requires meticulous planning when conducting simultaneous rhinoplasty and lip lift procedures [24,25]. Rhinoplasty modifies nasal structures, potentially affecting the nasolabial angle, which defines the nose-lip relationship. A lip lift enhances facial proportions by reducing upper lip length and improving lip shape, complementing the effects of rhinoplasty [10,20]. These combined surgeries demand coordinated planning to prevent facial feature imbalances. Scarring and healing are crucial factors to consider in these joint procedures [26]. Although both surgeries involve incisions that risk scarring, appropriate surgical methods can reduce visible marks. The studies examined in this analysis noted a low occurrence of hypertrophic scarring, with most scars fading within six months to one year. Functional outcomes were generally positive, with no reported long-term impairments in nasal airflow or lip movement. Temporary issues, such as mild numbness and swelling, were resolved in all instances. Despite concerns about vascular complications due to the abundant blood supply in the nose and lips, none were reported in the studies included in this analysis.

Rhinoplasty and lip lift procedures enhance facial harmony by reshaping the nose and reducing the space between the upper lip and nose base, refining the nasolabial angle and improving facial proportions [18,27]. These procedures are beneficial for individuals seeking feminization, as they contribute to a softer, more feminine appearance. Proper patient selection, preoperative counseling, and proficiency in both techniques are crucial for successful outcomes [28,29]. Preoperative planning, including 3D imaging and simulation, can help achieve desired results. Surgeons should minimize complication risks, particularly in postoperative scar management and infection prevention, and provide extended follow-up to monitor for potential late-onset complications.

Simultaneous rhinoplasty and lip lift surgery offer significant benefits for patients seeking improved facial harmony and aesthetic outcomes. The combination of these procedures is associated with high patient satisfaction and minimal complications. However, the success of these surgeries depends on careful patient selection, thorough preoperative planning, and the expertise of the surgeon. When performed together, rhinoplasty and lip lift provide a powerful approach to achieving superior aesthetic results in a wide range of patients.

While informative, this systematic review exhibits several constraints. The limited scope of five studies and 351 participants may restrict the broader applicability of findings. The diversity in patient profiles, surgical approaches, and follow-up periods introduces additional complexity. The variety of rhinoplasty and lip lift methods, coupled with auxiliary procedures such as forehead reconstruction or cheek augmentation, impedes definitive conclusions regarding the effects of combined surgeries. Moreover, the prevalence of female and transgender subjects in feminization procedures limits the applicability to the wider cosmetic facial surgery demographic. Another drawback is the absence of standardized outcome metrics. While some studies employed validated instruments like the Satisfaction with Aesthetic Results Scale (SGAIS), others relied on subjective patient feedback or clinical assessments, potentially introducing bias. The relatively short follow-up periods, spanning one to three years, may not capture long-term outcomes such as scar maturation or delayed complications. Furthermore, publication bias might skew results towards favorable outcomes, as studies with negative results may be less likely to be published. The dependence on retrospective studies, constrained by available data quality, presents an additional limitation. The lack of prospective studies with randomized controlled designs weakens the strength of conclusions. Variations in risk of bias assessment using the NOS indicate inconsistencies in study quality, necessitating caution when interpreting results.

This systematic review demonstrates numerous strengths despite its limitations. By following PRISMA guidelines, it ensures a methodical and open approach, bolstering the reliability of its conclusions. The review's thoroughness is enhanced by its incorporation of studies from various databases and the meticulous removal of duplicates, reducing the chance of overlooking pertinent research. The use of the Newcastle-Ottawa Scale for assessing bias adds validity, enabling a more precise interpretation of the results. A key strength lies in its concentration on the specific, under-researched area of concurrent rhinoplasty and lip lift procedures. By consolidating existing evidence, the review offers valuable insights into the aesthetic and functional outcomes of these combined surgeries, addressing a knowledge gap in the field. The consistent reporting of positive aesthetic results, high levels of patient satisfaction, and minimal complications across studies underscores the advantages of this integrated approach to facial enhancement. Examining complications and adverse events contributes to a better understanding of the risks associated with simultaneous surgery, helping clinicians to better inform patients and manage their expectations. Furthermore, the inclusion of diverse patient groups, including those undergoing gender-affirming procedures, broadens the review's applicability. The findings provide clinicians with crucial information on approaching combined facial surgeries in various demographics, particularly in facial feminization, where nasal and perioral features play a vital role in achieving satisfactory outcomes.

This review highlights areas for future investigation to enhance our understanding of combined rhinoplasty and lip lift procedures. To address the limitations of smaller retrospective analyses, larger, multi-institutional prospective studies with uniform outcome measures are essential for gathering robust data. Incorporating a wide range of patients, including both sexes and various ethnic groups, will improve the applicability of aesthetic results. The use of standardized outcome evaluations, such as 3D imaging or quantitative facial angle analysis, will allow for precise comparisons between surgical methods, elucidating the interplay between rhinoplasty and lip lift on facial aesthetics. Longer follow-up periods are necessary to assess the longevity of aesthetic outcomes, scar development, and delayed complications. Evaluating long-term functional results, including potential impacts on speech or facial expressions, is crucial for determining the safety of these combined surgeries. Additional research is required to examine how simultaneous rhinoplasty and lip lift operations affect overall satisfaction among transgender individuals, considering the unique aspects of gender-affirming procedures. Studies should also investigate novel surgical techniques or modifications to improve the safety and effectiveness of concurrent surgeries. Progress in minimally invasive methods, such as laser or endoscopic approaches, may reduce scarring and complications while enhancing results. Furthermore, exploring patient-specific factors like skin type, age, and initial anatomy could lead to more tailored treatment strategies.

## Conclusions

Combining rhinoplasty and lip lift procedures yields substantial cosmetic advantages and high patient contentment. This integrated approach improves facial balance, particularly regarding nasolabial and frontonasal angles, while enhancing lip proportions for a more harmonious overall appearance. The reviewed studies showed minimal complications, with mild scarring and temporary sensory alterations resolving within several months. Functional outcomes were positive, with no enduring impairments in nose or lip function. This systematic review indicates that the combined procedure is safe, efficient, and well-received, delivering enhanced aesthetic results and elevated patient satisfaction. Achieving optimal outcomes, especially in complex cases such as male-to-female gender confirmation surgeries, requires meticulous surgical planning, precise technique, and thorough postoperative care. This dual surgical method shows considerable promise for individuals seeking comprehensive facial enhancement.

## Appendices

**Table 4:** Search strings.

PubMed	(("Rhinoplasty" (MeSH Terms) OR "Rhinoplasty" (Title/Abstract) OR "simultaneous rhinoplasty" (Title/Abstract)) AND ("Lip" (MeSH Terms) OR "Lip Lift" (Title/Abstract) OR "Lip Surgery" (Title/Abstract)) AND ("Aesthetic Outcome"(Title/Abstract) OR "Esthetics" (MeSH Terms) OR "Cosmetic Outcome" (Title/Abstract)) AND ("Patient Satisfaction"(MeSH Terms) OR "Satisfaction" (Title/Abstract)))
Scopus	TITLE-ABS-KEY ("rhinoplasty" OR "simultaneous rhinoplasty") AND TITLE-ABS-KEY ("lip lift" OR "lip surgery") AND TITLE-ABS-KEY ("aesthetic outcomes" OR "cosmetic outcome" OR "esthetic outcome") AND TITLE-ABS-KEY ("patient satisfaction" OR "satisfaction")
Web of Science	TS= ("rhinoplasty" OR "simultaneous rhinoplasty") AND TS= ("lip lift" OR "lip surgery") AND TS= ("aesthetic outcomes" OR "cosmetic outcome" OR "esthetic outcome") AND TS= ("patient satisfaction" OR "satisfaction")
Medline	(rhinoplasty.mp. OR exp Rhinoplasty/ OR simultaneous rhinoplasty.mp.) AND (lip.mp. OR lip lift.mp. OR exp Lip/ OR lip surgery.mp.) AND (aesthetic outcomes.mp. OR exp Esthetics/ OR cosmetic outcome.mp.) AND (patient satisfaction.mp. OR exp Patient Satisfaction/)
Science Direct	("rhinoplasty" OR "simultaneous rhinoplasty") AND ("lip lift" OR "lip surgery") AND ("aesthetic outcomes" OR "cosmetic outcome" OR "esthetic outcome") AND ("patient satisfaction" OR "satisfaction")
